# Systematic and Quantitative Investigation of Newly Synthesized Proteins Reveals Distinct Ion Homeostasis and Mitochondrial Changes between Cuproptosis and Ferroptosis in Human Cells

**DOI:** 10.1021/acs.analchem.5c07257

**Published:** 2026-03-10

**Authors:** Yue Wu, Longping Fu, Xing Xu, Pak San Chan, Ronghu Wu

**Affiliations:** School of Chemistry and Biochemistry and the Petit Institute for Bioengineering and Bioscience, 1372 Georgia Institute of Technology, Atlanta, Georgia 30332, United States

## Abstract

Dysregulated metal ion metabolism and its connection to cell death attract great attention in cell biology and biomedicine. There are two major types of known metal ion-induced cell death so far. Well-documented ferroptosis is an iron-dependent form of cell death driven by lipid peroxidation, and the recently discovered cuproptosis is copper-dependent cell death, possibly related to mitochondrial damage and cell stress. Although some studies have suggested a possible link between cuproptosis and ferroptosis, the cellular responses and mechanistic differences between these two forms of metal ion-dependent cell death remain to be explored. Here, we systematically and quantitatively analyzed newly synthesized proteins (NSPs), which reflect rapid changes in gene expression, in cells undergoing ferroptosis and cuproptosis through integrating metabolic labeling, bioorthogonal chemistry, and multiplexed proteomics. The results revealed that both types of cell death shared some common features, such as mitochondrial disorder and gene expression suppression. Furthermore, different changes between them were also uncovered. In cuproptosis, proteins related to zinc ion homeostasis were elevated because intracellular copper and zinc ions are cooperatively and competitively involved in multiple biological processes, and excess copper ions impact zinc ion homeostasis in cells. Moreover, damaged mitochondria were found to be cleared mainly through ubiquitin-mediated mitophagy. In contrast, ferroptosis is associated with an increased level of calcium-binding proteins and a compensatory upregulation of key antioxidant defense systems while concurrently showing a notable decrease in RNA alternative splicing-related proteins. Taken together, a comprehensive and comparative analysis of NSPs in cuproptosis and ferroptosis provides us with a unique opportunity to understand the molecular mechanisms of these two important forms of metal ion-dependent cell death.

## Graphical abstract

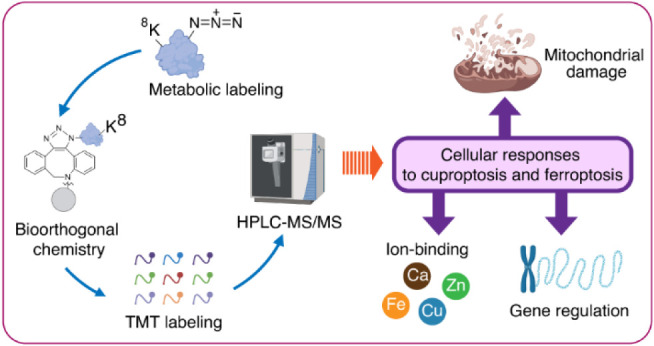

## Introduction

Regulated cell death (RCD) is a genetically controlled process essential for maintaining cellular and organismal homeostasis.^1^ It is strongly related to human diseases such as cancer, neurodegenerative disorders, and autoimmune diseases.^2,3^ Recent advances have expanded our understanding of RCD by uncovering new mechanisms closely tied to metabolic dysregulation.^4–6^ For instance, ferroptosis, an iron-dependent cell death, is driven by the accumulation of lipid peroxides,^7^ and this process involves reactive oxygen species (ROS) generated through the Fenton reaction under oxidative stress.^8^ More recently, cuproptosis was identified as a distinct copper-dependent RCD pathway. It was reported to be triggered when excess copper ions bind to lipoylated proteins in the mitochondrial tricarboxylic acid (TCA) cycle, leading to the aggregation of these proteins, depletion of iron–sulfur (Fe–S) cluster proteins, and subsequent proteotoxic stress.^9^ These two metal ion-associated RCD pathways highlight the importance of metal ion homeostasis in cells.

Emerging evidence indicates that ferroptosis and cuproptosis may be interlinked through their shared effects on mitochondrial function and the glutathione (GSH) antioxidant system.^10–12^ In cuproptosis, excessive copper ions target key TCA cycle enzymes such as DLAT, causing their aggregation,^9^ and erastin-induced ferroptosis leads to cysteine depletion, mitochondrial hyperpolarization, and lipid peroxide accumulation, accompanied by distinctive changes in mitochondrial morphology.^13^ A central protective mechanism in both cell death pathways is the GSH axis. GSH not only serves as a cofactor for Glutathione Peroxidase 4 (GPX4) to inhibit ferroptosis but also acts as a primary intracellular copper chelator to mitigate cuproptosis.^11,14^ Despite these insights, the cellular responses to ferroptosis and cuproptosis and their mechanistic differences remain to be explored. Most studies are focused on a set of core regulators, underscoring the need for systematic and quantitative analysis to uncover the shared and distinct molecular signatures of these metal ion-dependent cell death pathways.

Quantitative proteomics used in cell death studies is typically employed to quantify the abundance changes of proteins at the proteome level, providing a broad overview of cellular protein abundance changes.^9,14^ However, because total protein levels reflect the long-term balance between protein synthesis and degradation, this approach often fails to capture the rapid and transient protein changes in response to stress responses and during the death processes in cells. In contrast, systematic quantification of newly synthesized proteins enables us to uncover proteins actively produced in response to external stimuli and provides insights into dynamic cellular responses during RCD. Through metabolic labeling with amino acid analogs such as L-azidohomoalanine (AHA), combined with bioorthogonal chemistry, newly synthesized proteins (NSPs) can be selectively enriched. Then, they can be systematically quantified with high sensitivity because highly abundant corresponding existing proteins are removed.^15–17^ This strategy enables direct assessment of early regulatory and compensatory responses triggered by metal ion-induced stress in cuproptosis and ferroptosis. By capturing real-time translational reprogramming events, newly synthesized proteome analysis overcomes the limitations of conventional whole-proteome profiling and offers a more comprehensive molecular perspective on the onset and progression of regulated cell death.

In this study, we systematically investigated cellular responses to cuproptosis and ferroptosis by integrating metabolic labeling, bioorthogonal chemistry, and quantitative multiplexed proteomics. This strategy enabled us to systematically quantify NSPs during metal ion-induced cell death. At the same time, we also quantified the abundance changes of total proteins at the proteome level, and as expected, the newly synthesized proteome is markedly more dynamic than the total proteome in both cuproptosis and ferroptosis. The results revealed both shared and distinct features among newly synthesized proteins in cuproptosis and ferroptosis. One common feature was a pronounced reduction in histones, indicative of reduced gene expression during these reprogrammed cell deaths. During cuproptosis, it was found to uniquely activate a strong zinc-associated response with decreased synthesis of Fe–S cluster proteins involved in electron transfer and to preferentially engage the ubiquitin-mediated mitophagy pathway to remove damaged mitochondria. Nuclear adaptation during cuproptosis was characterized by increased levels of DNA methylation-related proteins and the activation of transcription factors with monomeric or homomultimeric binding modes. In contrast, during ferroptosis, we observed increased levels of calcium-binding proteins and a compensatory upregulation of antioxidant defenses, accompanied by a decrease in the abundance of RNA splicing-related proteins. Collectively, these findings help distinguish ferroptosis and cuproptosis through their specific and dynamic protein expression signatures, thereby advancing the mechanistic understanding of metal ion-induced cell death.

## Experimental Section

### Cell Culture and Treatment, and Measurement of Cell Viability

MCF7 cells (American Type Culture Collection, ATCC) were maintained in Dulbecco’s Modified Eagle Medium (DMEM; Sigma-Aldrich) supplemented with 10% fetal bovine serum (FBS; Phoenix Scientific) and 1% penicillin–streptomycin (Sigma-Aldrich). Cells were cultured at 37 °C in a humidified incubator containing 5% CO_2_. For the cell viability assay experiment, 5 × 10^3^ cells were seeded per well in 96-well plates and treated with varying concentrations of erastin (MedChemExpress) or elesclomol (MedChemExpress) in the presence of 1 μM CuSO_4_, and the control samples were treated with 0.5% DMSO. For the zinc supplementation experiments, cells were cotreated with 20 μM or 50 μM ZnSO_4_ to evaluate its effect on cell survival. The cell viability was determined using the Cell Counting Kit-8 (CCK-8; MedChemExpress), and the results were expressed as the relative viability normalized to the control.

### Metabolic Labeling and Protein Extraction

MCF7 cells were cultured in T75 flasks until reaching ∼80% confluence and then treated with 15 μM erastin or 5 nM elesclomol in the presence of 1 μM CuSO_4_ for 24 h, or with 0.5% DMSO as a control. We performed biologically triplicate experiments. During the treatment, cells were incubated in lysine- and methionine-depleted DMEM supplemented with 10% dialyzed FBS (Fisher Scientific) for 0.5 h, maintaining the same concentrations of the respective cell death inducers or DMSO. Cells were then metabolically labeled with heavy lysine (^13^C^6^ and ^15^N^2^, + 8 Da; Lys8, Cambridge Isotope Laboratories) and AHA (Vector Laboratories) for 4 h. The labeling medium contained the same concentrations of heavy lysine and AHA as lysine and methionine in standard DMEM (0.8 mM and 0.2 mM, respectively) and was supplemented with the same concentrations of each cell death inducer or DMSO. After labeling, cells were harvested using a cell scraper and pelleted by centrifugation at 300 × g for 5 min. The pellets were washed twice with ice-cold PBS and lysed through end-over-end rotation at 4 °C for 1 h in the lysis buffer containing 50 mM 4-(2-hydroxyethyl)­piperazine-1-ethanesulfonic acid (HEPES, pH = 8.3), 150 mM NaCl, 0.5% sodium deoxycholate (SDC), 25 units/mL Benzonase Nuclease (Sigma-Aldrich), and the cOmplete protease inhibitor cocktail (Sigma-Aldrich). The lysates were clarified by centrifugation at 4,696 × g for 10 min at 4 °C. Protein concentrations were measured using the Pierce BCA Protein Assay Kit (Thermo Fisher Scientific). Ninety percent of each sample was used for newly synthesized protein analysis, and the remaining 10% was for total proteome analysis.

### Newly Synthesized Protein Enrichment

Dibenzocyclooctyne (DBCO)-conjugated magnetic beads were prepared as described previously.^15–17^ Briefly, 800 μL of amine-derivatized MagnaBind beads (Fisher Scientific, ∼10 μmol -NH_2_) were washed three times with 1.5 mL of anhydrous DMSO to remove residual aqueous buffer. The beads were then resuspended in anhydrous DMSO to a final volume of 2 mL, supplemented with 40 μL of N,N-diisopropylethylamine (DIPEA, Sigma-Aldrich), and preincubated for 30 min with constant rotation. Subsequently, DBCO-sulfo-*N*-hydroxysuccinimide ester (DBCO-sulfo-NHS; 12 μmol; Vector Laboratories) was added. The reaction was allowed to proceed at room temperature overnight in the dark to ensure maximal conjugation. The DBCO-functionalized beads were washed three times with 2 mL of 50% acetonitrile (ACN) to remove unreacted reagents and then stored in 1.6 mL of 20% ACN at 4 °C for further use. Before enrichment, the beads were thoroughly washed with the cell lysis buffer. Equal amounts of total proteins were then transferred to new tubes and incubated with the DBCO-modified beads at 4 °C overnight.

### Protein Digestion, Peptides Labeled with TMT, and Fractionation by HPLC

Enriched newly synthesized proteins or total proteins in the whole proteome experiment were reduced with 5 mM dithiothreitol (DTT) at 56 °C for 25 min, followed by alkylation with 15 mM iodoacetamide (IAA) at room temperature for 30 min in the dark. Residual IAA was quenched by the addition of 10 mM DTT for 15 min. For the enriched samples, the beads were then subjected to sequential washes with 2 mL of lysis buffer containing 2.5% sodium dodecyl sulfate (SDS) and 2.5% sodium deoxycholate (SDC) four times at 95 °C, 2 mL of 8 M urea in 100 mM HEPES (pH 8.3) three times, 2 mL of 50% isopropanol twice, and 2 mL of 50% ACN twice. After washing, the magnetic beads were resuspended in the digestion buffer (50 mM HEPES, pH 8.3, 1.6 M urea), and NSPs were digested overnight at 37 °C with lysyl endopeptidase (Lys-C; Wako) at an enzyme-to-protein ratio (w/w) of 1:100 under gentle shaking. For the whole proteome samples, proteins were precipitated using the methanol/chloroform/water method in a ratio of 4:1:3. The mixture was centrifuged at 4696 g for 10 min. The resulting protein pellet was air-dried and subsequently dissolved in buffer with 1.6 M urea and 50 mM HEPES (pH 8.3) for digestion. Digestion was performed overnight at 37 °C using sequencing-grade modified trypsin (Promega) at an enzyme-to-protein ratio (w/w) of 1:100.^18,19^ This ratio allows for more complete protein digestion while minimizing trypsin autolysis. The digestion was terminated by adding trifluoroacetic acid (TFA) to a final concentration of 0.4%. Samples were centrifuged to remove precipitates and desalted using 50 mg SepPak tC18 cartridges (Waters).

Purified peptides were dried and then dissolved in 35 μL of 100 mM HEPES (pH 8.5) containing 10 μL of ACN, followed by labeling with the TMT10plex reagents (control: 126, 127N, 127C; erastin treatment: 128N, 128C, 129N; elesclomol and 1 μM CuSO_4_ treatment: 129C, 130C, 131; Thermo Fisher Scientific), respectively. After labeling for 1 h, the reaction was quenched by adding 4 μL of 10% hydroxylamine. Peptides from each experimental group were combined, cleaned, and fractionated by high-pH reversed-phase HPLC into 24 fractions using a 40-min gradient from 5% to 50% ACN in 10 mM ammonium formate (pH 10). The collected fractions were purified using stage tips and dried in a speed vac prior to LC-MS/MS analysis.

### LC-MS/MS Analysis

Dried peptides were resuspended in the loading solution containing 5% ACN and 4% formic acid (FA), and 4 μL was loaded onto a microcapillary column packed with C18 beads (ReproSil-Pur 120 C18-AQ, 1.9 μm, Dr. Maisch). Peptides were separated using a nanoflow reversed-phase HPLC (Ultimate 3000 RSLC, Dionex) with a 97-min gradient of 2 to 30% ACN (with 0.125% FA) designed for TMT-labeled peptides. The full MS and MS2 were recorded using an Orbitrap Exploris 480 Mass Spectrometer (Thermo Scientific). Each cycle included one full MS scan (resolution: 120,000, scan range: *m*/*z* 350–1400) in the Orbitrap at the standard automatic gain control (AGC) target, followed by data-dependent MS/MS (ddMS2) scans for the most intense ions. MS2 spectra were acquired in the Orbitrap at a resolution of 15,000 with an isolation window of 0.7 *m*/*z*. Higher-energy collisional dissociation (HCD) was applied at a normalized collision energy of 38%. The normalized AGC target was set to 200%, and the maximum injection time was 100 ms.

### Database Searching, Data Filtering, and Protein Quantification

MS raw files were searched using SEQUEST^20^ against the *Homo sapiens* UniProt database (https://www.uniprot.org/taxonomy/9606). The search parameters for NSPs were set as follows: precursor mass tolerance, 10 ppm; product ion mass tolerance, 0.025 Da; enzyme, Lys-C (up to three missed cleavages); and a maximum of four variable modifications per peptide. Variable modifications include methionine oxidation (+15.9949 Da) and heavy lysine (+8.0142 Da), while fixed modifications are cysteine carbamidomethylation (+57.0214 Da) and TMT labeling on lysine residues and peptide N-termini (+229.1630 Da). For the whole proteome analysis, the following search parameters were employed: precursor mass tolerance, 10 ppm; product ion mass tolerance, 0.025 Da; enzyme, Trypsin (up to three missed cleavages); and a maximum of four variable modifications per peptide. Variable modifications include methionine oxidation (+15.9949 Da), while fixed modifications include cysteine carbamidomethylation (+57.0214 Da) and TMT labeling on lysine residues and peptide N-termini (+229.1630 Da). Peptide-spectrum matches were filtered using the target-decoy strategy to achieve <1% false discovery rate (FDR) at the peptide level. The FDR was further controlled to <1% at the protein level. After that, only peptides with XCorr (>1.2), S/N (>5), and precursor mass error (<10 ppm) were kept, and contaminated peptides were removed. For the NSP results, an additional filter was applied to include only peptides labeled with heavy lysine (+8.0142 Da). The TMT reporter ion intensities from MS2 spectra were used for protein quantification. For each protein, the summed intensities of all unique peptides were calculated. The average of triplicate samples was used to determine the protein abundance ratio between the treated and control (DMSO) samples.

### Bioinformatic Analysis

Over-representation analysis of proteins was performed using clusterProfiler (v4.12.6) with the Gene Ontology database.^21^ Terms with *P* < 0.05 and fold change (Treatment/Control) > 1.30 or <0.77 were considered significant. Nuclear-related proteins were annotated using the EpiFactors database (epifactors.autosome.org). The workflow and nuclear event illustrations were created using BioRender (biorender.com) and the chemical structures were produced using ChemDraw (v23.1.1). Data visualization and statistical analyses were conducted in GraphPad Prism 10.0 and R Studio (v4.5).

## Results

### Quantification of Newly Synthesized Proteins and the Whole Proteome in Cells with Cuproptosis and Ferroptosis

To systematically investigate cellular responses to cuproptosis and ferroptosis, we designed a quantitative proteomic workflow to globally and quantitatively study newly synthesized proteins by combining metabolic labeling, bio-orthogonal chemistry, and multiplexed proteomics (Figure 1A). To determine the appropriate treatment conditions to induce cuproptosis and ferroptosis in cells, different concentrations of elesclomol with 1 μM CuSO_4_ and erastin were used, respectively. Following the measurement of cell viability over a 24-h period, we selected the IC20 concentration of each inducer for subsequent proteomic experiments (Figure 1B and C). Based on previous work,^22–24^ the IC20 concentrations were chosen to capture cell-death-associated cellular responses while maintaining sufficient protein synthesis for NSP analysis. A 24-h treatment time point was used because it is commonly regarded in cell death studies as a stage at which cells are actively engaging in death programs rather than merely exhibiting sublethal stress responses.^9,25^ While treated with the inducers, cells were subjected to methionine and lysine starvation, followed by labeling with AHA and heavy lysine. Next, AHA-labeled proteins were selectively captured using the DBCO-conjugated magnetic beads (Figure S1A) through bioorthogonal chemistry. Subsequently, on-bead protein digestion was performed, and peptides were labeled with the TMT-10plex reagents, respectively. All samples were then pooled, fractionated, and subjected to LC-MS analysis.

**Figure 1:**
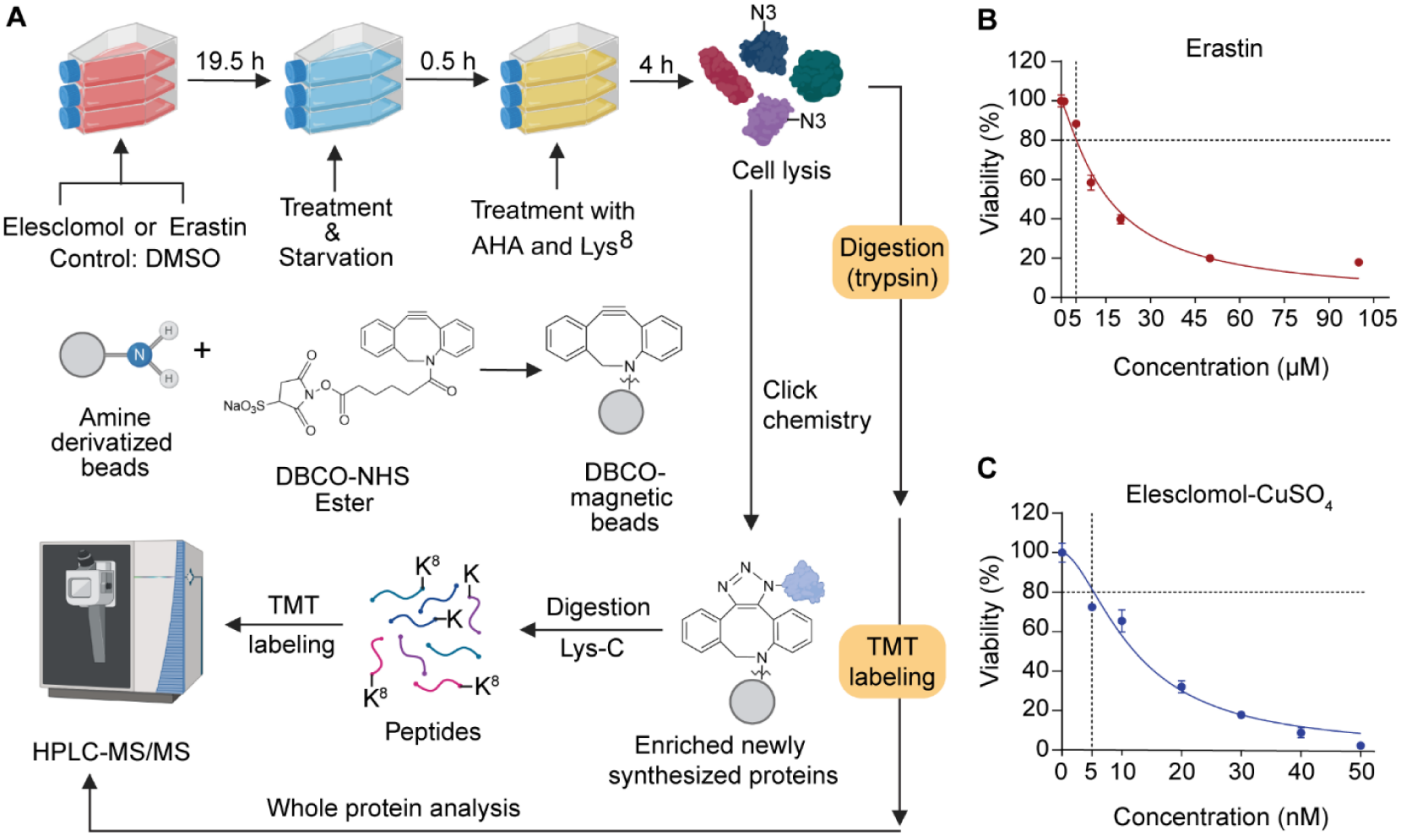
**Overview of newly synthesized protein analysis in cells with cuproptosis and ferroptosis.** (A) Experimental workflow for inducing cell death and enriching newly synthesized proteins using the DBCO-magnetic beads. (B) The cell viability after incubation with the indicated concentrations of Erastin for 24 h in each group (*n* = 4). (C) The cell viability after incubation with the indicated concentrations of elesclomol and 1 μM CuSO_4_ for 24 h in each group (*n* = 4). Data are means of triplicate samples ± standard deviation (SD), and the error bars indicate the SD.

We quantified 6,630 NSPs and 8,314 proteins in the whole proteome (Tables S1 and S2). The distribution of the NSP abundance changes was significantly broader than that of the whole proteome (Figure 2A). Besides, a notable difference was observed for quantified NSPs in cells between cuproptosis and ferroptosis, while not for proteins in the whole proteome. For example, as shown in Table 1, HSPA1B (Fold Change = 0.47) and CLGN (Fold Change = 0.48) exhibited significant downregulation, whereas their corresponding proteins in the whole proteome showed only minor changes (Fold Change = 0.85 and 0.79, respectively). This is expected because, in the whole proteome experiment, there are many existing copies of corresponding proteins, which make overall protein ratios closer to 1. NSPs are much more sensitive and can be indicative of real cellular responses compared to those of their corresponding proteins in the whole proteome.

**Figure 2:**
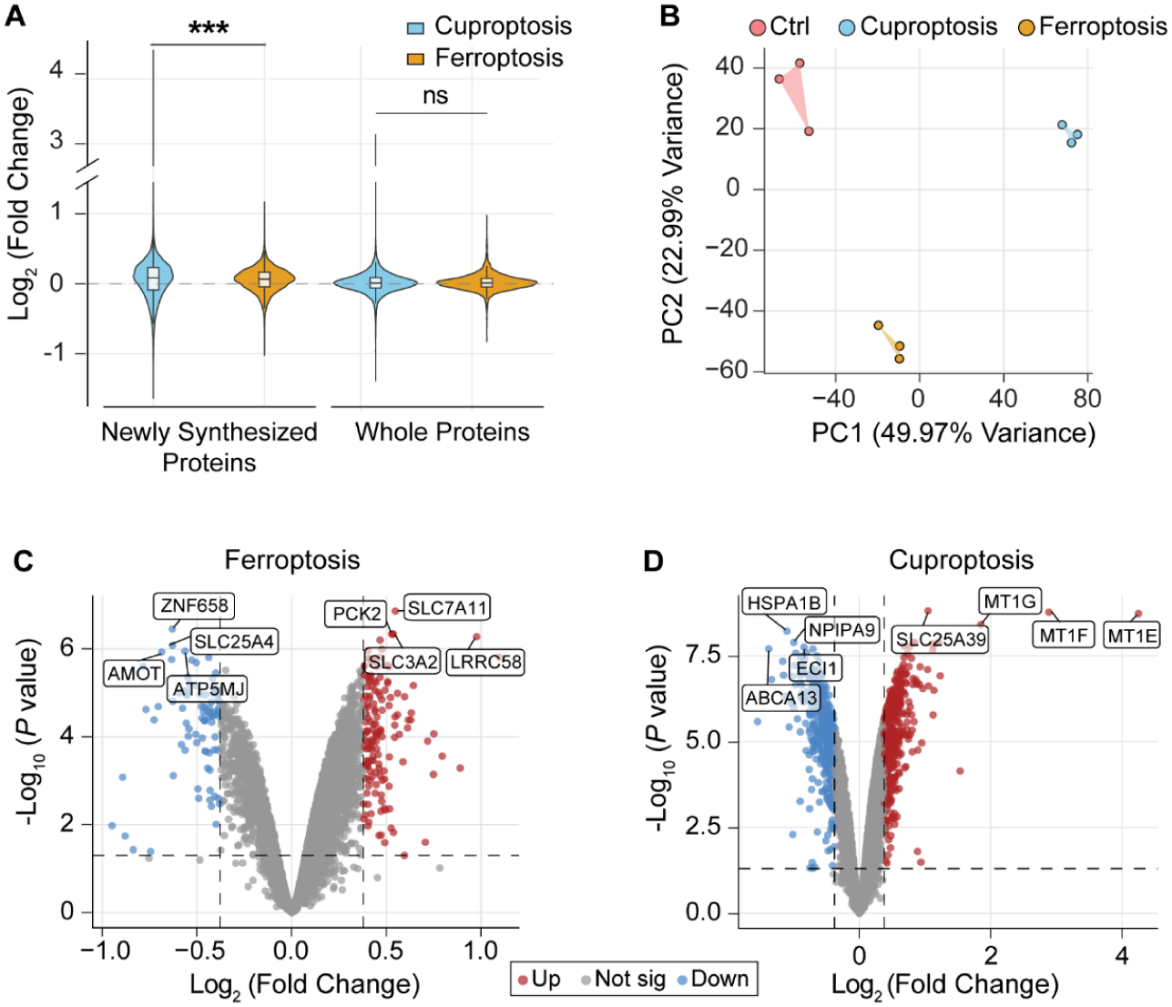
**Overall analysis of total proteins and newly synthesized ones in cuproptosis and ferroptosis.** (A) Overall ratio distributions of total proteins and newly synthesized ones in cuproptosis and ferroptosis. (B) Principal component analysis of all samples (triplicate samples for cuproptosis, ferroptosis, and control, respectively). (C–D) Volcano plots of Log_2_(fold change) vs -Log_10_(*p*-value) show the differentially expressed proteins in (C) ferroptosis and (D) cuproptosis (*p*-value < 0.05 and fold change > 1.30 or <0.77). Box: 25th/75th percentiles; center line: mean; whiskers: 1.5-fold the interquartile range (IQR). The differences were assessed using the two-sided Mann–Whitney U test: ***p < 0.001.

**Table 1:** Abundance Changes of Representative NSPs and Total Proteins (WP) in Cells with Cuproptosis Compared to Those in the Control Samples

Gene name	Fold change in NSP	Fold change in WP	Annotation
HSPA1B	0.47	0.85	Aiding protein folding and preventing aggregation
CLGN	0.48	0.79	Calcium-binding protein
ATP5IF1	0.49	0.74	Inhibiting ATP synthase in the mitochondrion
ECI1	0.56	0.82	Key enzyme of fatty acid beta-oxidation
DYNC1H1	1.64	1.07	Transport of vesicles and organelles
TRIM21	1.64	1.12	E3 ubiquitin-protein ligase
ASNS	1.76	1.26	Amino-acid biosynthesis
SLC30A1	1.95	1.47	Transporting Zn^2+^ out of cell

The principal component analysis (PCA) results show clear separation among the cuproptosis, ferroptosis, and control groups, and the clustering of biological triplicates for each group (Figure 2B), clearly demonstrating that the current results are highly reproducible. In ferroptosis, we observed the upregulation of SLC7A11 and SLC3A2 (Figure 2C), and these two proteins were previously reported as key regulators of antioxidant defense/System Xc^–^ in ferroptosis, particularly in response to erastin-induced cell death.^26^ Because erastin triggers ferroptosis by blocking the function of SLC7A11, the upregulation of these proteins is expected as cells respond to ferroptotic stress.^27^ In cuproptosis, 524 NSPs were upregulated, while 430 were downregulated (Figure 2D). Among the upregulated ones, those belonging to the metallothionein-1 (MT1) family exhibited the highest abundance changes, indicating the effect of cuproptosis on metal binding and the cellular detoxification process.

To understand the relationship between cuproptosis and ferroptosis, we compared the differentially expressed NSPs under each condition (Figure 3A). The results showed that 67 proteins were commonly upregulated in both cuproptosis and ferroptosis, while 51 proteins were downregulated (Table S3). The Gene Ontology (GO) analysis of the shared upregulated proteins revealed strong enrichment in ion binding, carbohydrate derivative binding, lipid biosynthetic processes, response to oxygen-containing compounds, and the oxoacid metabolic process (Figure 3B). Conversely, the commonly downregulated NSPs were predominantly associated with chromosome organization, cytoskeleton regulation, DNA binding, chromatin remodeling, and cellular component organization. These findings indicate that both types of cell death have shared features, i.e., the suppression of gene expression and cellular component organization.

**Figure 3:**
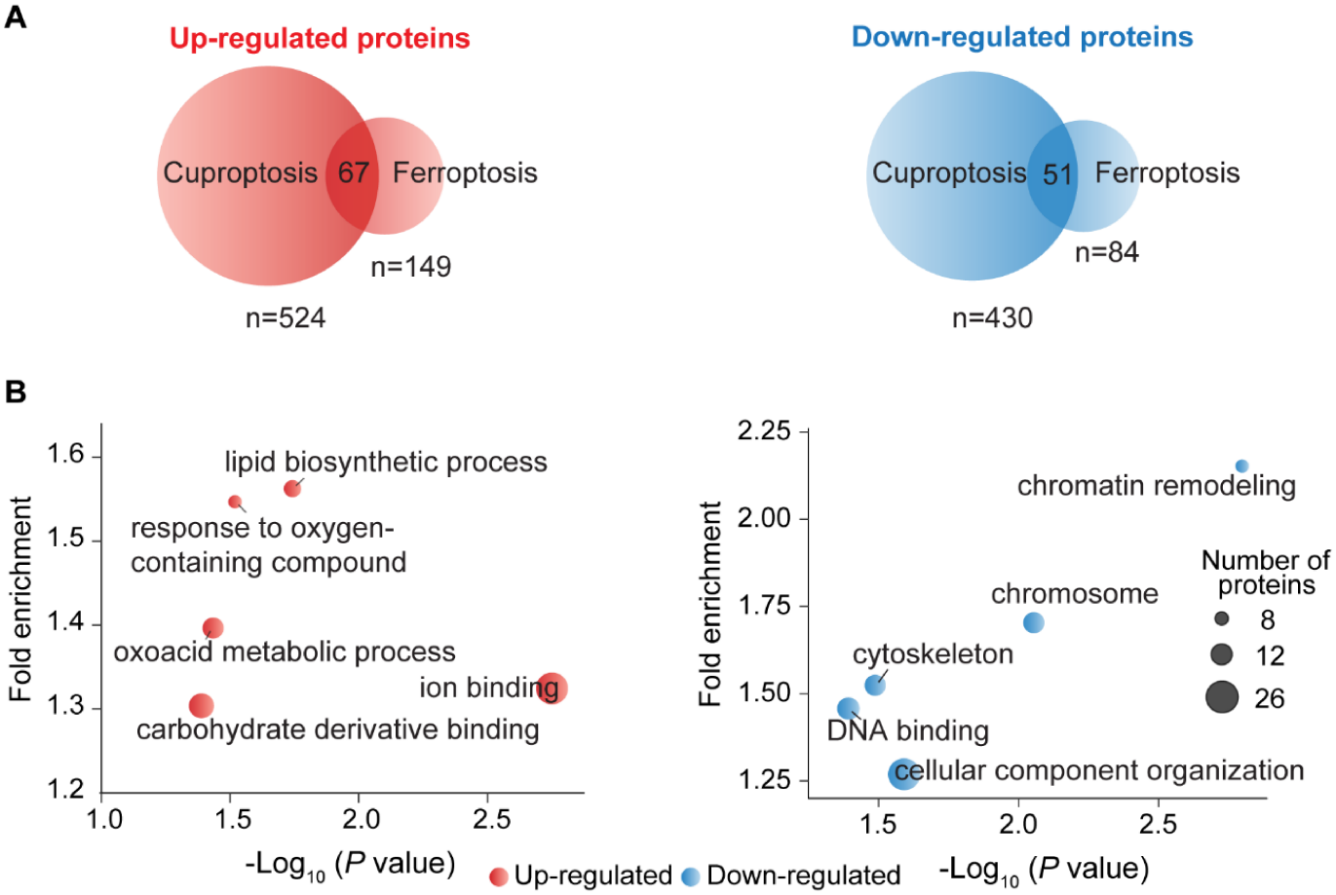
**Evaluation of the unique and common differentially expressed proteins in cuproptosis and ferroptosis.** (A) Overlap of the differentially expressed proteins in cuproptosis and ferroptosis. (B) GO analysis of the common differentially expressed proteins in both the cuproptosis and ferroptosis groups.

### Investigation of Ion-Binding Proteins during Cuproptosis and Ferroptosis

Given that both cuproptosis and ferroptosis are closely associated with metal ion metabolism and ion binding (Figure 3B), we further investigated the NSPs related to ion binding under these two cell death conditions (Figure 4A). The results revealed that while there are no dramatic differences in copper- and iron-binding proteins between cuproptosis and ferroptosis, the changes in calcium- and zinc-binding proteins differed significantly. Ferroptosis involves plasma membrane rupture and allows extracellular calcium ions to flood the cytosol. The elevated abundance of calcium-binding proteins may be a stress response to this calcium influx.^28^ While ferroptosis was characterized by an increase in calcium-binding proteins, the abundance of zinc-binding proteins showed a significant increase in the cuproptosis group (Figure 4A). Zinc-binding proteins can be categorized into constitutive and inducible subgroups.^29^ Constitutive zinc-binding proteins are normally stabilized by zinc ions, and inducible zinc-binding proteins are dynamic sensors of free zinc ion levels. In our results, the abundances of both constitutive and inducible zinc-binding proteins were significantly increased in cuproptosis compared to ferroptosis, indicating a broad activation of zinc-associated responses (Figure 4B). Copper and zinc are both essential intracellular metal ions involved in multiple biological processes. They contribute to cellular homeostasis as cofactors and dynamically interact through both cooperative and competitive mechanisms.^30,31^ Therefore, we also investigated the regulation of NSPs related to zinc ion homeostasis. The results showed that the proteins in the MT1 family, including MT1E, MT1F, and MT1G, were significantly upregulated. These specific proteins are known to bind both zinc and copper ions under normal conditions (Figure 4C).^30^ Similarly, the exporter ZNT1 was upregulated, while the zinc ion importer SLC39A6 was downregulated. These results suggest a potential regulatory response of zinc homeostasis and a cellular effort to modulate the metal-binding capacity as a defense against copper toxicity.

**Figure 4:**
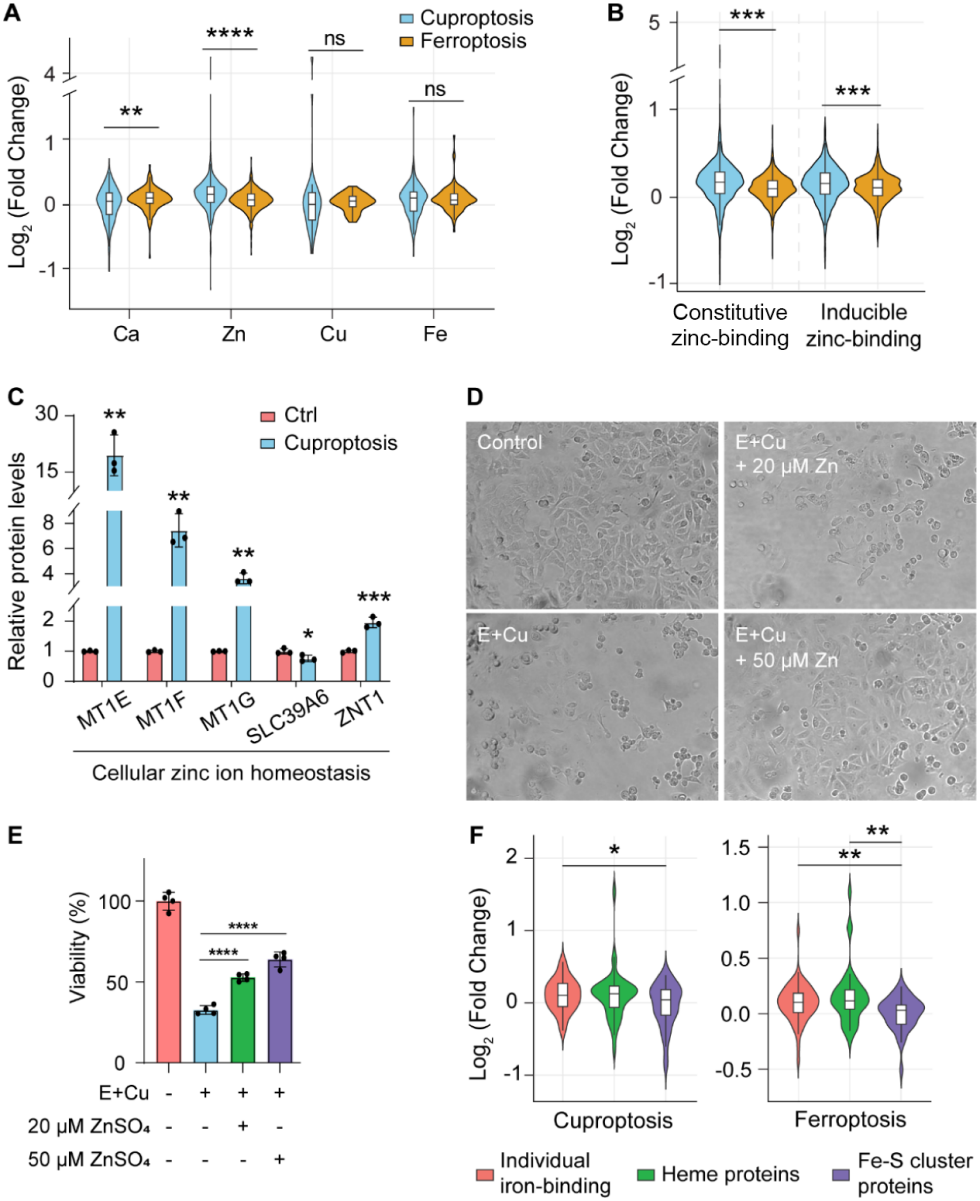
**Investigation of ion-binding proteins (NSPs) during cuproptosis and ferroptosis.** (A–B) Changes in metal-binding proteins (A), and regulation of constitutive and inducible zinc ion-binding proteins (B) in cuproptosis and ferroptosis. (C) Relative protein levels in cellular zinc ion homeostasis. (D–E) Representative morphological changes (×100) (D), and viability of MCF7 cells (E) treated with 0.5% DMSO (Control) or 20 nM Elesclomol+1 μM CuSO_4_ (E+Cu) in the presence or absence of ZnSO_4_ (20 or 50 μM). Data are means of triplicate samples ± SD, and the error bars indicate SD. (F) Differential regulation of iron ion-binding proteins in cuproptosis and ferroptosis. Box: 25th/75th percentiles; center line: mean; whiskers: 1.5-fold IQR. The differences were assessed using the two-sided Student’s *t*-test for (C and E) and Mann–Whitney U test for other comparisons: *p < 0.05, **p < 0.01, ***p < 0.001, and ****p < 0.0001.

Given that the current proteomic data revealed an active cellular response to regulate zinc homeostasis during cuproptosis, we further investigated whether zinc ion levels directly influence cuproptosis-induced cytotoxicity. By treating MCF7 cells with the cuproptosis inducer, we observed significant cell death and morphological distress (Figure 4D, E+Cu). Strikingly, the addition of exogenous zinc ions effectively rescued the cells from cuproptosis in a dose-dependent manner. Representative images showed that cells maintained a more normal morphology with increasing concentrations of zinc ions (Figure 4D). This protective effect was further confirmed by a cell viability assay, which demonstrated a significant increase in survival rates as the zinc ion concentration was increased from 20 to 50 μM (Figure 4E). These findings indicate that zinc ions may exert a protective role against copper-induced toxicity, further reinforcing the functional interplay between these two essential metal ions. The functional significance of zinc in modulating copper-induced toxicity is also supported by its clinical application in Wilson’s disease (WD), a hereditary disorder characterized by copper accumulation, where zinc ion supplementation serves as an effective therapeutic method for WD.^32^

Because the aggregation and destabilization of Fe–S cluster proteins were previously reported as features of cuproptosis,^9^ we next explored how these proteins changed among NSPs.^33^ While individual iron-binding proteins and heme proteins generally showed an upward trend in cuproptosis and ferroptosis, Fe–S cluster proteins exhibited a less significant change in cuproptosis compared to those in ferroptosis (Figure 4F). However, the levels of newly synthesized Fe–S cluster proteins involved in the electron transfer process^33^ showed a significant decrease during cuproptosis but remained stable in ferroptosis (Figure S2). In the mitochondrion, Fe–S cluster proteins are an essential component of the electron transport chain (ETC) complexes, particularly Complex I (NDUFS1, NDUFV1, and NDUFV2) and Complex II (SDHB).^34^ The reduction of electron-transferring Fe–S cluster proteins represents a key feature in cuproptosis and explains the subsequent reduction of mitochondrial respiratory capacity.^9^

### Mitochondrial Damage and Clearance, and Redox Remodeling in Cuproptosis and Ferroptosis

The mitochondrion is central to energy production and cell death control. It possesses a double-membrane structure consisting of an outer membrane, an intermembrane space, an inner membrane, and a matrix enriched with metabolic enzymes.^35^ Investigation of mitochondrial proteins provides insights into how different cell death pathways affect mitochondrial functions. Both ferroptosis and cuproptosis are closely linked to mitochondrial dysfunction, and they have been reported to affect mitochondrial metabolism and integrity through distinct mechanisms.^9,11,13^ Analysis of mitochondrial NSPs in cuproptosis and ferroptosis revealed that commonly upregulated proteins were predominantly localized in the matrix, including key metabolic enzymes ALDH1L2 and PCK2, as well as the lipid transporter PRELID3B (Figure 5A and Table S4, protein annotation from MitoCoP^36^). Their increased abundance suggests enhanced metabolic activity and stress adaptation in the mitochondrion.

**Figure 5:**
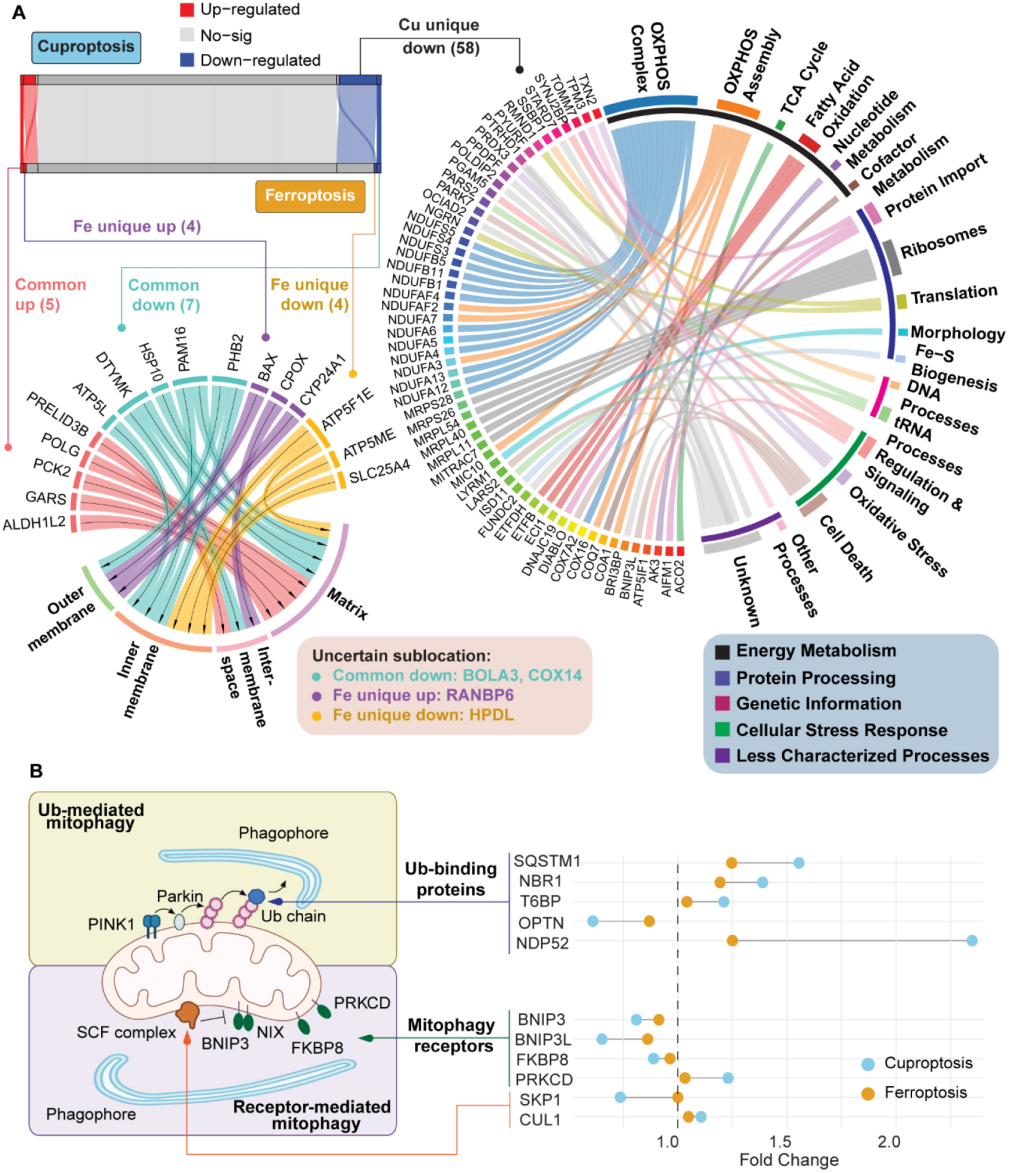
**Common and distinct regulation of mitochondrial proteins and the mitophagy pathways in cuproptosis and ferroptosis.** (A) Localization and functional analysis of common and unique mitochondrial proteins. Up-regulated: *p*-value < 0.05 and fold change > 1.30; Down-regulated: *p*-value < 0.05 and fold change < 0.77; No-sig: all other features. (B) Response of proteins in the mitophagy pathways in cuproptosis and ferroptosis.

We also found that several mitochondrial proteins were consistently downregulated in both cuproptosis and ferroptosis (Figure 5A). For example, ATP5L and COX14 are critical components of the oxidative phosphorylation machinery,^37^ and their downregulation indicates a reduction in ATP production. BOLA3 is essential for Fe–S cluster assembly,^38^ and its reduced level may disrupt mitochondrial enzyme function. Collectively, the downregulation of these proteins shows a broad impairment of mitochondrial bioenergetics under both forms of cell death. Beyond the shared protein regulations, mitochondrial localization analysis based on the MitoCoP database^36^ reveals that similar compartment changes of mitochondrial NSPs happened in both cuproptosis and ferroptosis. In both conditions, proteins of the outer membrane showed an upward trend, possibly reflecting increased stress signaling or changes in organelle communication. In contrast, the abundances of the inner membrane proteins generally decreased. The results are consistent with a reduction in oxidative phosphorylation components and impaired respiratory function in ferroptosis and cuproptosis (Figure S3).

Given the observed mitochondrial damage, we next examined the clearance of damaged mitochondria through mitophagy, a process mediated by both ubiquitin-dependent and receptor-dependent pathways.^39,40^ In cuproptosis, we observed a pronounced activation of the ubiquitin-mediated mitophagy pathway. The levels of key autophagy adaptor proteins, including SQSTM1, NBR1, T6BP, and NDP52, significantly increased, with NDP52 showing the most prominent increase (Figure 5B).^40^ These adaptors are known to bind ubiquitinated mitochondrial substrates and recruit the upstream autophagy machinery to initiate phagophore formation around damaged mitochondria. Mechanistically, this pathway is typically triggered by PINK1 accumulation on the outer mitochondrial membrane and subsequent activation of the E3 ligase Parkin, which together generate ubiquitin chains to serve as molecular “eat-me” signals for adaptor recruitment.^40^ Meanwhile, receptor-mediated NSPs in the mitophagy pathway appeared to be suppressed under cuproptosis. The levels of canonical mitophagy receptors located on the mitochondrial outer membrane, such as BNIP3, BNIP3L (NIX), and FKBP8, all decreased (Figure 5B). These receptors normally act as direct links between damaged mitochondria and the autophagy machinery, initiating mitophagy independently of ubiquitin signaling.^39^ Collectively, these results reveal the selective removal of damaged mitochondria through ubiquitin-mediated mitophagy during cuproptosis.

Mitochondrial dysfunction is closely related to cellular redox balance, and damage to the mitochondria often induces oxidative stress. To further dissect the downstream consequences of cuproptosis and ferroptosis, we studied the responses of NSPs involved in redox regulation. In the lipid oxidation pathway, enzymes catalyzing the activation of polyunsaturated fatty acids (PUFAs) to PUFA-CoA, such as ACSL1 and ACSL4,^8^ were markedly upregulated under cuproptosis, showing enhanced lipid metabolism and potential oxidative stress. In contrast, ferroptosis caused a disruption of the antioxidant defense system, primarily targeting the GSH-GPX4 axis. GPX4 is the key enzyme responsible for detoxifying lipid peroxides, and its activity relies on GSH to prevent lipid accumulation.^41^ Erastin induces ferroptosis by depleting GSH, thereby directly resulting in GPX4 inactivation.^42^ Because the biosynthesis of GSH requires cystine as an essential precursor, cells may enhance the uptake of these amino acids to replenish GSH under oxidative stress. Accordingly, the levels of NSPs in cystine transporters SLC7A11 and SLC3A2 were markedly increased (Figure S4). This pattern is consistent with previous findings that cells upregulated SLC7A11 to compensate for the inhibition of cystine import by erastin.^43^ In addition, HSPA5, a member of the Hsp70 family known to stabilize GPX4,^44^ was also elevated, suggesting a coordinated attempt to preserve redox homeostasis.

### Gene Regulation and DNA-Associated Responses in Cuproptosis and Ferroptosis

Cell death often affects nuclear processes and gene regulation. Histones define chromatin structure and control DNA accessibility (Figure 6A).^45^ Histones can be classified into replication-dependent and replication-independent types.^46^ The abundance of newly synthesized histones decreased substantially in both cuproptosis and ferroptosis, with a more pronounced reduction observed for replication-dependent histones (Figure 6B). Replication-dependent histones are synthesized during the S phase to support nucleosome formation and maintain genome integrity during DNA replication. Their reduction indicates the inhibition of DNA synthesis and cell proliferation under both cell death conditions.

**Figure 6:**
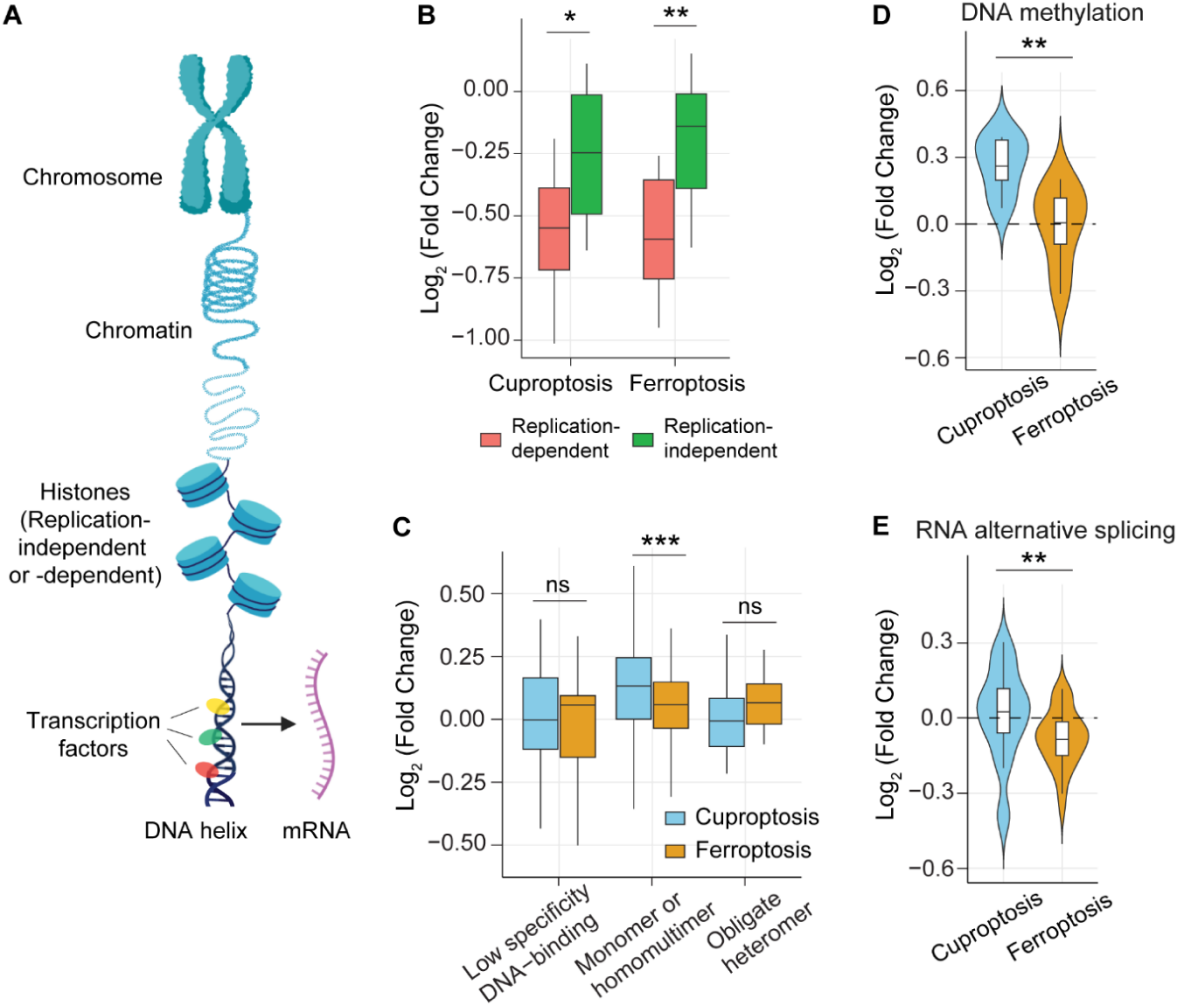
**Analysis of chromosome and gene expression in cuproptosis and ferroptosis.** (A) Schematic illustrating the process of gene transcription. (B) Changes in the levels of replication-dependent and -independent histones in cuproptosis and ferroptosis. (C–E) Analysis of changes in transcription factors with different binding modes (C), NSPs related to DNA methylation (D), and NSPs related to RNA alternative splicing (E) during cuproptosis and ferroptosis. Box: 25th/75th percentiles; center line: mean; whiskers: 1.5-fold IQR. The differences were assessed using the two-sided Mann–Whitney U test: *p < 0.05, **p < 0.01, and ***p < 0.001.

Furthermore, we analyzed transcription factors based on their DNA-binding modes. Three major categories are low-specificity DNA binding, monomer/homomultimer binding, and obligate heteromer binding.^47^ Among them, the abundance of those transcription factors with the monomer/homomultimer binding mode showed a significant increase in cuproptosis compared to ferroptosis (Figure 6C). These transcription factors can bind DNA directly without partner proteins and respond rapidly to stress, enabling quick activation of target genes under cellular stress. This enhanced binding mode suggests an immediate transcriptional response that may compensate for energy imbalance and promote adaptive gene expression. In the downstream process, transcription factors regulate the flow of genetic information from DNA to RNA (Figure 6A). We found that the levels of proteins involved in DNA methylation were elevated in cuproptosis but remained largely unchanged in ferroptosis (Figure 6D), whereas those associated with RNA alternative splicing showed little change in cuproptosis but decreased markedly during ferroptosis (Figure 6E).^46^ DNA methylation regulates chromatin compaction and transcriptional silencing, while alternative splicing generates multiple transcript isoforms from the same gene.^48^ Enhanced DNA methylation may stabilize the chromatin structure under copper-induced stress, while the reduction in splicing activity during ferroptosis suggests a global suppression of post-transcriptional regulation in ferroptosis.

## Discussion

Cuproptosis and ferroptosis represent two major types of metal ion-associated cell death pathways that disrupt cellular homeostasis through metal ion-induced stress.^11,12^ While previous studies relied on measuring the abundance changes of total proteins, dynamic changes in protein production were often masked. In this work, by combining metabolic labeling, bioorthogonal chemistry, and multiplexed proteomics, we systematically and quantitatively analyzed newly synthesized proteins in cuproptosis and ferroptosis. The current method allows us to better understand the mechanisms of different types of cell death that are often obscured by the large pool of preexisting proteins. As shown above, the abundance changes of NSPs were much more sensitive to cellular stress than those of total proteins. Although both cell death processes are featured by metal ion dysregulation, mitochondrion disorder, and oxidative stress, cuproptosis and ferroptosis represent two mechanistically distinct modes of regulated cell death.

In elesclomol-induced cuproptosis, the inhibition of ETC Complexes I and II has been shown to protect cells from copper toxicity.^9^ Our proteomic analysis revealed a pronounced decrease in the number of newly synthesized Fe–S cluster proteins involved in electron transfer during cuproptosis. The reduced level of synthesis of these ETC-associated Fe–S proteins may represent an adaptive response that limits respiratory activity, thereby mitigating further copper-induced mitochondrial damage. In this context, the decreased levels of those Fe–S cluster proteins could function as a cellular defense mechanism to counteract cuproptosis. Nevertheless, as previously reported, even though downregulation of various components in Complex I/III can delay cuproptosis, sufficient intracellular copper accumulation ultimately triggers cell death.^14^ Thus, while transient suppression of Fe–S protein synthesis may provide temporary protection, excessive copper levels eventually overwhelm these compensatory responses.

Such mitochondrial impairment typically activates cellular quality control systems to remove damaged organelles and restore homeostasis.^39^ Consistent with this cellular response, the ubiquitin-mediated mitophagy pathway was found to be strongly activated during cuproptosis. This preference for ubiquitin-dependent rather than receptor-mediated mitophagy may arise from the specific features of copper-induced mitochondrial damage. Because ubiquitin-dependent mitophagy is commonly triggered by the loss of mitochondrial membrane potential, this finding implies that cuproptosis may involve mitochondrial depolarization.^40,49^ While both cuproptosis and ferroptosis are due to metal-ion-driven toxicity, our comparative analysis of NSPs revealed distinct metal ion regulations. Cuproptosis induced increased levels of zinc-binding proteins, indicating enhanced zinc-associated stress responses. Concurrently, zinc homeostasis was actively regulated, as the zinc ion exporter ZNT1 was upregulated, while the zinc ion importer SLC39A6 was downregulated. These coordinated changes in zinc transporters are consistent with a cellular response to the altered zinc homeostasis. Potentially elevated availability of zinc can activate metal-binding proteins such as members of the MT1 family.^50^ These metal-binding proteins may reduce copper ion toxicity during the detoxification process.^51^ Accordingly, the observed zinc-related responses may contribute to buffering copper-induced toxicity. Although these changes raise the possibility that dysregulated zinc homeostasis represents an adaptive component of the cellular response to cuproptosis, they may also reflect a broader response to metal-induced stress. Further investigation, including direct measurements of intracellular free zinc flux, would be necessary to determine the specificity and mechanistic relevance of this response.

Epigenetic regulation plays an important role in shaping the cellular response to metal ion-induced stress. Previous studies indicated minimal nuclear morphological changes during ferroptosis but showed chromatin fragmentation during cuproptosis.^52,53^ Nonetheless, the current results show a shared consequence in these two cell death pathways: they both caused a global reduction of histones, particularly replication-dependent histones, pointing directly to cell-cycle arrest and inhibition of DNA synthesis. However, there are some differences between the two types of cell death. Analysis of downstream DNA-binding transcription factors revealed increased levels of those NSPs with the monomer-homomultimer binding mode in cuproptosis. This trend may reflect fast and independent transcriptional responses during cuproptosis.^47^ Furthermore, we found that DNA methylation-related NSPs were elevated in cuproptosis and a decrease in RNA alternative splicing-related NSPs was observed in ferroptosis. Our newly synthesized proteomic data revealed different epigenetic adjustments between these two types of metal ion-induced cell death. The increased abundance of DNA methylation-related proteins may reflect enhanced DNA methylation, a mediator of transcriptional silencing leading to the downstream gene expression downregulation.^54^ During cuproptosis, this epigenetic modification likely correlates with our observation of large-scale protein synthesis reduction, specifically the downregulation of various mitochondrial proteins. Simultaneously, enhanced DNA methylation activity may serve as a protective response to reinforce chromatin stability,^55^ potentially acting as a defense mechanism against replication stress in cuproptosis. Consistently, previous studies identified DNA methylation as both a prognostic marker and an epigenetic stabilizer in cuproptosis.^56^ On the other hand, the reduction of splicing-related NSPs in the context of erastin-induced ferroptosis could destabilize the cellular splicing network, hindering RNA processing and maturation.^57^ This disruption leads to proteomic imbalance that potentially impairs critical biological processes, including cellular growth and differentiation.^58^ Given the close relationship between ferroptosis and redox stability, dysregulated alternative splicing events may shift the expression of key antioxidant enzymes or metabolic regulators.^59^ These splicing-driven alterations may ultimately disrupt the cellular redox balance and modulate susceptibility to ferroptosis. Ferroptosis-related alternative splicing signatures were reported as potential biomarkers for prognosis and therapeutic response in cancer.^60^ Together, these findings highlight metal ion-dependent epigenetic regulation as a key mechanism linking cell death pathways to disease progression and potential therapeutic intervention.

In summary, this study provides a comprehensive comparison of cellular responses in cuproptosis and ferroptosis through global and quantitative analysis of newly synthesized proteins. The results reveal shared and distinct responses in these two important types of metal ion-induced cell death, and mitochondrial disorder and gene expression suppression occurred in both cuproptosis and ferroptosis. Although both cuproptosis and ferroptosis involve mitochondrial dysfunction and oxidative stress, they cause fundamentally different responses. Cuproptosis triggers zinc-associated stress responses, activation of ubiquitin-mediated mitophagy, and enhanced chromatin stabilization through DNA methylation, whereas ferroptosis is characterized by an active response in antioxidant defense and reduced splicing activity. These findings deepen our understanding of the molecular mechanisms in metal ion-associated cell death, leading to the development of new strategies to manipulate programmed cell death and disease treatment in the future.

## Supplementary Material











## Data Availability

The mass spectrometry proteomics data has been deposited to MassIVE (massive.ucsd.edu) with the dataset identifier MSV000099889.

## Notes

Supporting figures showing the schematic of click chemistry-based NSP enrichment (Figure S1); comparative analysis of electron transfer-related iron–sulfur cluster proteins (Figure S2), mitochondrial components (Figure S3); and redox homeostasis (Figure S4) in cuproptosis and ferroptosis (PDF)
Quantitative proteomics data for newly synthesized proteins (Table S1) (XLSX)
Whole proteome analysis during cuproptosis and ferroptosis (Table S2) (XLSX)
List of commonly regulated NSPs across both cell death pathways (Table S3) (XLSX)
Mitochondrial annotation of NSPs using the MitoCoP database (Table S4) (XLSX)
