# Supplementary material for: Systematic and Quantitative Investigation of Newly Synthesized Proteins Reveals Distinct Ion Homeostasis and Mitochondrial Changes between Cuproptosis and Ferroptosis in Human Cells
Source: Anal Chem. 2026 Mar 10;98(11):8309–20. doi: 10.1021/acs.analchem.5c07257 (PMC13019430; doi:10.1021/acs.analchem.5c07257)
Supplement: Supplementary file 1 [file ac5c07257_si_001.pdf]

Supporting Information

Systematic and Quantitative Investigation of Newly Synthesized Proteins Reveals Distinct Ion Homeostasis and Mitochondrial Changes between Cuproptosis and Ferroptosis in Human Cells

Yue Wu, Longping Fu, Xing Xu, Pak San Chan, and Ronghu Wu*

School of Chemistry and Biochemistry and the Petit Institute for Bioengineering and Bioscience, Georgia Institute of Technology, Atlanta, Georgia 30332, USA

* Correspondence: ronghu.wu@chemistry.gatech.edu (R.W.)

This PDF file includes:

Figures S1 to S4

Other supporting materials for this manuscript include the following:

Table S1 to S4

Table S1. Quantification of newly synthesized proteins in cells with cuproptosis and ferroptosis

Table S2. Quantification of proteins in cells with cuproptosis and ferroptosis in the whole proteome experiment

Table S3. List of commonly regulated newly synthesized proteins in cuproptosis and ferroptosis

Table S4. Mitochondrial annotation of newly synthesized proteins using MitoCoP

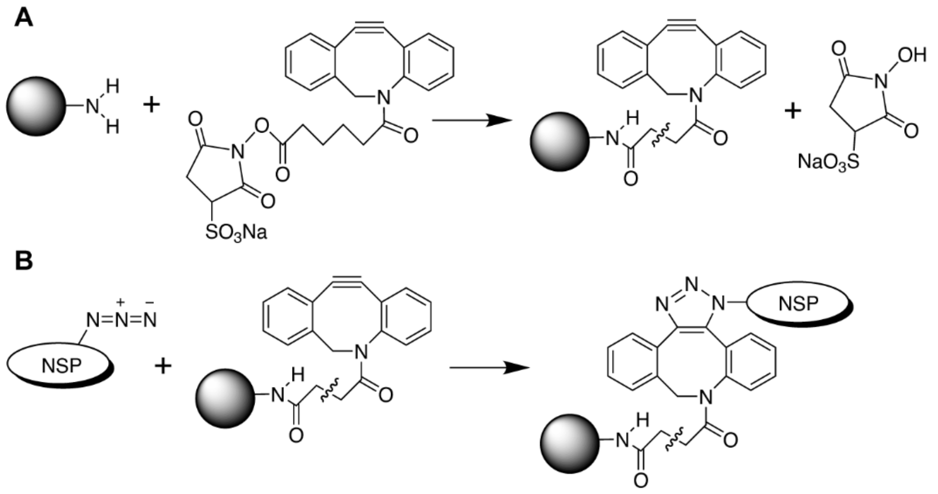

Figure. S1. Click chemistry-based enrichment of newly synthesized proteins. A) Bead synthesis for the click chemistry-based method to enrich newly synthesized proteins. B) Newly synthesized proteins labeled with L-Azidohomoalanine are captured using click chemistry.

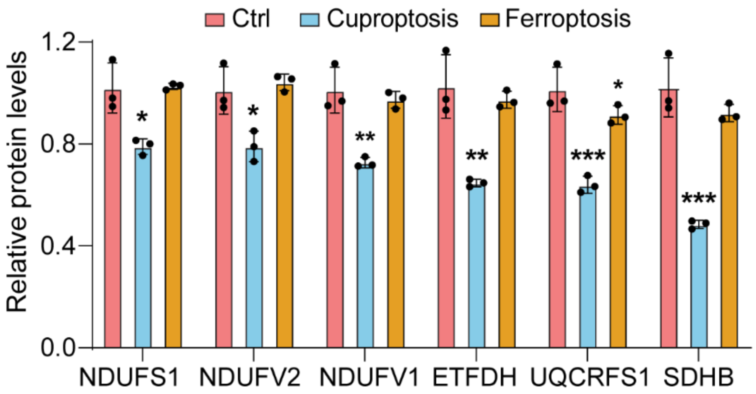

Figure. S2. Differential regulation of electron transfer-related Fe-S cluster proteins in cuproptosis and ferroptosis. Data are means of triplicate samples ± standard deviation (SD) and the error bar is SD. The differences were assessed using the two-sided Student's t-test: *$p < 0.05$, **$p < 0.01$ and ***$p < 0.001$.

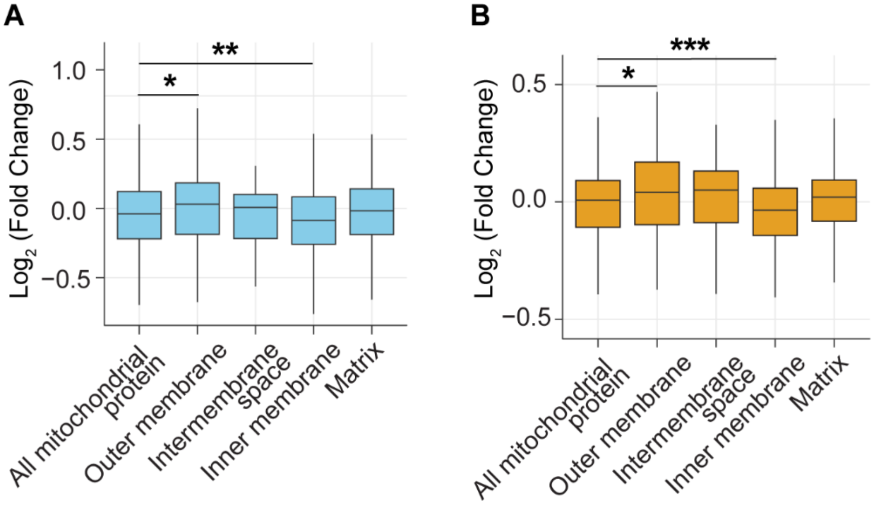

Figure. S3. Comparative proteomic analysis of mitochondrial and redox systems in cuproptosis and ferroptosis. A-B) Distributions of NSPs in different mitochondrial sublocations in A) cuproptosis and B) ferroptosis. Box: 25th/75th percentiles; center line: mean; whiskers: 1.5-fold the interquartile range (IQR). The differences were assessed using the two-sided Mann-Whitney U test: *$p < 0.05$, **$p < 0.01$ and ***$p < 0.001$.

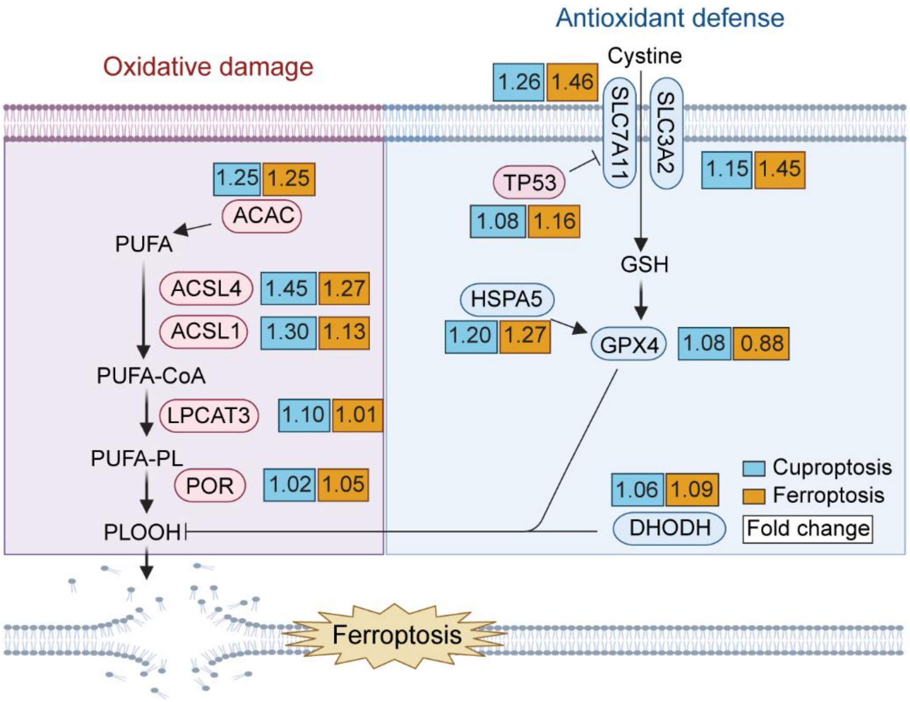

Figure. S4. Comparative analysis of the redox system in cuproptosis and ferroptosis.
